# GINS2 attenuates the development of lung cancer by inhibiting the STAT signaling pathway

**DOI:** 10.7150/jca.46744

**Published:** 2021-01-01

**Authors:** Dianmin Sun, Yuanyuan Zong, Jinling Cheng, Zhenxiang Li, Ligang Xing, Jinming Yu

**Affiliations:** 1 Department of Thoracic Surgery, Shandong Cancer Hospital Affiliated to Shandong University, Shandong Academy of Medical Sciences, Jinan, Shandong 250117, China; 2 Department of Pathology, Shandong Provincial Hospital Affiliated to Shandong University, Jinan, Shandong 250117, China; 3 Department of Gastroenterology, Shandong Provincial Western Hospital, Jinan, Shandong 250117, China; 4 Department of Radiation Oncology, Shandong Cancer Hospital Affiliated to Shandong University, Shandong Academy of Medical Sciences, Jinan, Shandong 272173, China; 5 Shandong University, Jinan, Shandong 250117, China

**Keywords:** GINS2, STAT, lung cancer, proliferation, apoptosis

## Abstract

GINS complex subunit 2 (GINS2) controls DNA replication. GINS2 expression is upregulated in several kinds of aggressive tumors. However, the effect of GINS2 in lung cancer remains unclear. We performed TCGA database analysis to confirm the clinical significance of GINS2 in lung cancer. After silencing GINS2 in A549 cells, we performed MTT assays, flow cytometry assays, colony formation assays, cell cycle analyses and RNA sequence analysis to elucidate the effect of GINS2 on lung cancer. Moreover, we assessed tumor growth and analyzed body fluorescence in mice as a measure of tumor burden. The TCGA database analysis demonstrated that GINS2 mRNA and protein was highly expressed in three kinds of lung cancer tissues. Subsequently, knockdown of GINS2 inhibited cell proliferation, colony formation, cell cycle arrest and apoptosis in A549 cells. On the other hand, we also investigated the effect of GINS2 on tumor formation in vivo. The analysis of nude mouse tumors showed that the tumor volume and weight of shGINS2 mice were significantly smaller than those of the control mice. To reveal the mechanism of GINS2 in lung cancer, we collected A549 cells with GINS2 knockdown to examine the downstream gene expression changes. The results showed that STAT1 and STAT2 mRNA and protein expression were significantly upregulated after GINS2 knockdown in A549 cells. Our results suggest that GINS2 inhibits the proliferation of lung cancer cells by inhibiting the STAT signaling pathway, which may be a potential biomarker for the diagnosis or prognosis of lung cancer.

## Introduction

Lung cancer is the leading cause of cancer-related death worldwide, and more than 85% of lung cancer patients suffer from non-small-cell lung cancer (NSCLC) 1, 2. Despite the improvement of treatment methods, the long-term survival rate of NSCLC remains low due to cancer recurrence and metastasis, with a 5-year survival rate of less than 15% 3-5. Therefore, new prognostic biomarkers and therapeutic targets need to be determined urgently.

GINS complex family members were named GINS complex subunit (GINS), including GINS2, GINS3 and GINS4. GINS complex family members play an essential role in the initiation of DNA replication and the cell cycle 6. In addition, GINS2 may play an important role in cell division, specifically by promoting chromosome segregation 7. It has been reported that GINS2 is involved in the tumorigenesis of various cancers, and its molecular weight is approximately 21 kDa 8. Specifically, GINS2 was found to be highly expressed in colorectal cancer by genome-wide gene expression profile analysis 9. In addition, GINS2 is related to the invasive characteristics of breast cancer, and it is presumed that GINS2 is related to the metastasis of lung cancer 10. Furthermore, the enhanced expression of GINS2 can promote the proliferation of leukemia cells and desensitize them to apoptosis 11. These findings suggest that GINS2 may play a central role in tumorigenesis.

In the previous study, Chi et al discovered that knockdown of GINS2 inhibits proliferation and promotes apoptosis through the p53/growth arrest and DNA damage 45A pathway in NSCLC 12. Additionally, another research group performed the experiment to demonstrate that GINS2 facilitates epithelial-to-mesenchymal transition in NSCLC cancer through modulating phosphoinositide-3-kinase/protein kinase B and mitogen-activated protein kinase/extracellular signal-regulated kinase pathway 13. In present study, we determined the clinical significance of GINS2 in lung cancer and inhibited the proliferation of lung cancer cells by inhibiting the signal transducer and activator of transcription (STAT) signaling pathway. In addition, we have gained a new insight into the important role of GINS2 in NSCLC development.

## Materials and Methods

### Cell culture

Human embryonic kidney 293 (HEK293), human MCF-7 breast cancer cells, human umbilical vein endothelial cell (HUVECs), human SW480 colon cancer cells, 95D, A549, NCI-H1299 and NCI-H1975 cells were purchased from American Type Culture Collection (ATCC) and grown adherently in a humidified incubator at 37°C with 5% CO_2_. All cells were maintained in Dulbecco's modified Eagle's medium (DMEM) high glucose containing 10% fetal bovine serum (FBS), penicillin (100 U/mL) and streptomycin (100 mg/mL). The medium was changed every two days.

### Construction of the GINS2 shRNA Lentiviral Vector

The GINS2 shRNA vector was constructed with a lentiviral plasmid. The GINS2 (GeneBank accession number: NM_016095) and scrambled shRNA oligonucleotides are listed in supplementary table 1. They were inserted into pLentiHI plasmids at AgeI and EcoRI sites and confirmed by sequencing. The 8.9 packaging plasmid and VSVG envelope protein plasmid were combined with the shRNA empty vector and then cotransfected into HEK293T packaging cells using Lipofectamine 2000 (Invitrogen, USA) 14. After transfection for 48 h, the supernatant was collected and filtered through a 0.45 μm filter to remove cellular debris to collect the virus particles.

A549 cells (1×10^6^/mL) were cultured in 6 well plates and infected with 4 µL lentivirus. The virus titer was 5×10^8^ TU/mL. The expression of green fluorescent protein was observed after transfection for 72 h by fluorescence microscopy. The experiment was divided into two groups, shGINS2 and shCtrl.

### Animal models

Eight-week-old male BALB/c nude mice were purchased from Beijing Vital River Laboratory Animal Technology Company and randomly divided into two groups, negative control (NC): GFP shRNA; and knockdown (KD): GINS2 shRNA, with 10 mice in each group. The protocol was performed according to a previous study 15. We subcutaneously injected 5×10^6^ A549 cells with shGINS2 or shCtrl lentivirus into the right flank of each mouse. Tumor size was measured every four days after injection, and then we calculated tumor volume according to the formula: volume= length×width^2^×3.16/6. Animal care and experimental procedures were approved by the Animal Care and Use Committee of Shandong University.

### RNA extraction and real-time PCR

We extracted total RNA from A549 cells or mouse tissues using RNAiso Plus (Invitrogen, USA) and immediately performed RT-PCR to synthesize cDNA using PrimeScript™ RT Master Mix (Takara, Japan). qRT-PCR was performed using specific primers and SYBR Green (Invitrogen, USA) on Applied Biosystems (Thermo, USA). qRT-PCR was performed in triplicate to amplify specific genes and normalize them to β-actin levels. Primer sequences are listed in supplementary table 2.

### Differentially expressed gene analysis

We performed the experiment according to a previous study 16. First, clean reads were obtained from the results. Then, we aligned the clean reads with the human reference genome (GCF_000001405.38) using TopHat. Cufflinks was used to normalize the gene expression in RPKM (reads per million per kilobase). We used DESeq software to analyze the differentially expression genes to compare the NC and KD groups. The false discovery rate (FDR) was used to set the significance threshold of the *p*-value in multiple tests. The significance of gene expression was judged by FDR ≤ 0.05 and absolute value of fold change ≥ 2 as the thresholds.

### Western blotting

Western blotting was performed as previously described 17. Briefly, cells were lysed and separated on 10% SDS-polyacrylamide gels. The following primary antibodies (Abs) were used: GINS2, B-cell lymphoma-2 (Bcl-2), Bax, caspase-3, cyclin dependent kinase inhibitor 1a (CDKN1A), CDKN1B, cyclin dependent kinases 4 (CDK4), cyclin D, bone morphogenetic protein receptor type 2 (BMPR2), interferon induced protein with tetratricopeptide repeats 1 (IFIT1), STAT1, STAT2, interferon regulatory factor 1 (IRF-1), tumor necrosis factor alpha induced protein 3 (TNFAIP3), interferon induced transmembrane protein 1 (IFITM1) and glyceraldehyde-3-phosphate dehydrogenase (GAPDH). Signals were detected using the Immobilon reagent (Millipore, USA) and visualized using a Bio-Rad ChemiDoc XRS (Bio-Rad, USA). Visualized signal intensities were quantitatively analyzed using Quantity One software (Bio-Rad, USA). All primary Abs were purchased from Cell Signaling Technology (Beverly, USA).

### Cell growth assay

A549 cells were seeded into 96-well plates at a density of 5×10^3^ cells/well. We transfected shCtrl or shGINS2 lentivirus into A549 cells during the logarithmic growth phase. We counted the cells daily using the Celigo Imaging Cytometer (Nexcelom Bioscience, USA) and repeated the experiment three times.

### MTT cell proliferation assay

A549 cells were seeded into 96-well plates at a density of 5×10^3^ cells/well, and cell viability was assessed using MTT (Thermo Fisher, USA). We added the MTT solution and incubated it for 4 h at 37°C. Then, we added 10 μL dimethyl sulfoxide to form formazan crystals and mixed for 3 min. Then, we measured the absorbance at 490 nm by using a microplate reader (Tecan Infinite, Austria). The MTT assay was repeated three times.

### Colony formation assay

A549 cells were seeded in six-well plates at 10^5^ cells/well, and the medium was changed every two days and cultured in 5% CO_2_, 37°C. We transfected the lentivirus shGFP and shGINS2 into A549 cells and observed the status of the cells. After being washed with phosphate-buffered saline (PBS), cells were fixed with 4% paraformaldehyde for 30 min. Then, the cells were stained with Giemsa staining solution for 8 min and washed 3 times with ddH_2_O. We counted and photographed the colonies under a microscope (Nikon, Japan).

### Cell cycle analysis

Flow cytometry analysis was used to detect cell cycle distribution. A549 cells were transfected with shGFP or shGINS2 lentivirus. Then, the cells were collected and washed with prechilled D-Hanks solution (pH 7.2) and fixed for 1 h in prechilled 75% ethanol. Cells were stained with a solution containing D-Hanks solution, propidium iodide (Sigma, USA) and RNase concentrate (10 mg/mL) (1000:25:10) and analyzed on a flow cytometer (Guava^®^ easyCyte Flow Cytometers, USA). Cell cycle analysis was performed in triplicate.

### Cell migration assay

Cell migration was detected using a wound-induced migration assay 18. In brief, A549 cells were seeded in 6 well plates at 1×10^5^ cells/well. Once the cells grew and reached 80% confluence, a straight line was scraped with a pipette tip to create a “scratch”, and the cells were washed 3 times with PBS. We replaced the medium with medium specific for the in vitro scratch assay. The wound healing images were captured at 0 and 48 h post-wounding under a Nikon microscope (Nikon, Japan). The ratio of the cell recovery area was measured to evaluate cell migration.

### Immunohistochemistry

For the microscopic quantification of the GINS2 expression, the lung cancer tissue was cut into 8-10 sections as previously described 15. To microscopic evaluation of GINS2 expression in the lung cancer tissues at different stages, serial paraffin sections of the lung cancer tissue were immunohistochemical (IHC) stained with the GINS2 antibodies (Sigma, USA). Secondary antibodies included anti-Goat IgG for GINS2 staining (Beijing Zhongshan Biotechnology, China).

Two independent observers blinded to the histopathological features and patient data of the samples evaluated and scored the degree of immunostaining. The scores were based on the proportion of positively stained tumor cells [graded as: negative (=0% positive), week (1-25% positive), positive (26-50% positive) or strong positive (>51% positive)] and staining intensity [categorized as 1 (no staining), 2 (weak staining, light yellow), 3 (moderate staining, yellow brown) or 4 (strong staining, brown)].

### The Cancer Genome Atlas (TCGA) data analysis

GINS2 profiles of 515 lung adenocarcinoma tissues and 59 lung tissues were downloaded from TCGA database (http://genome-cancer.ucsc.edu) and used to determine the correlation among the GINS2 expression, clinicopathological features and prognosis of patients with lung adenocarcinoma.

### Statistical analysis

The statistical analyses were carried out by one-way ANOVA followed by LSD test using GraphPad 7.0 software. In all cases, data were expressed as the mean ± SEM. *P*<0.05 was considered statistically significant.

## Results

### GINS2 was highly expressed in lung cancer specimens

**Figure 1:**
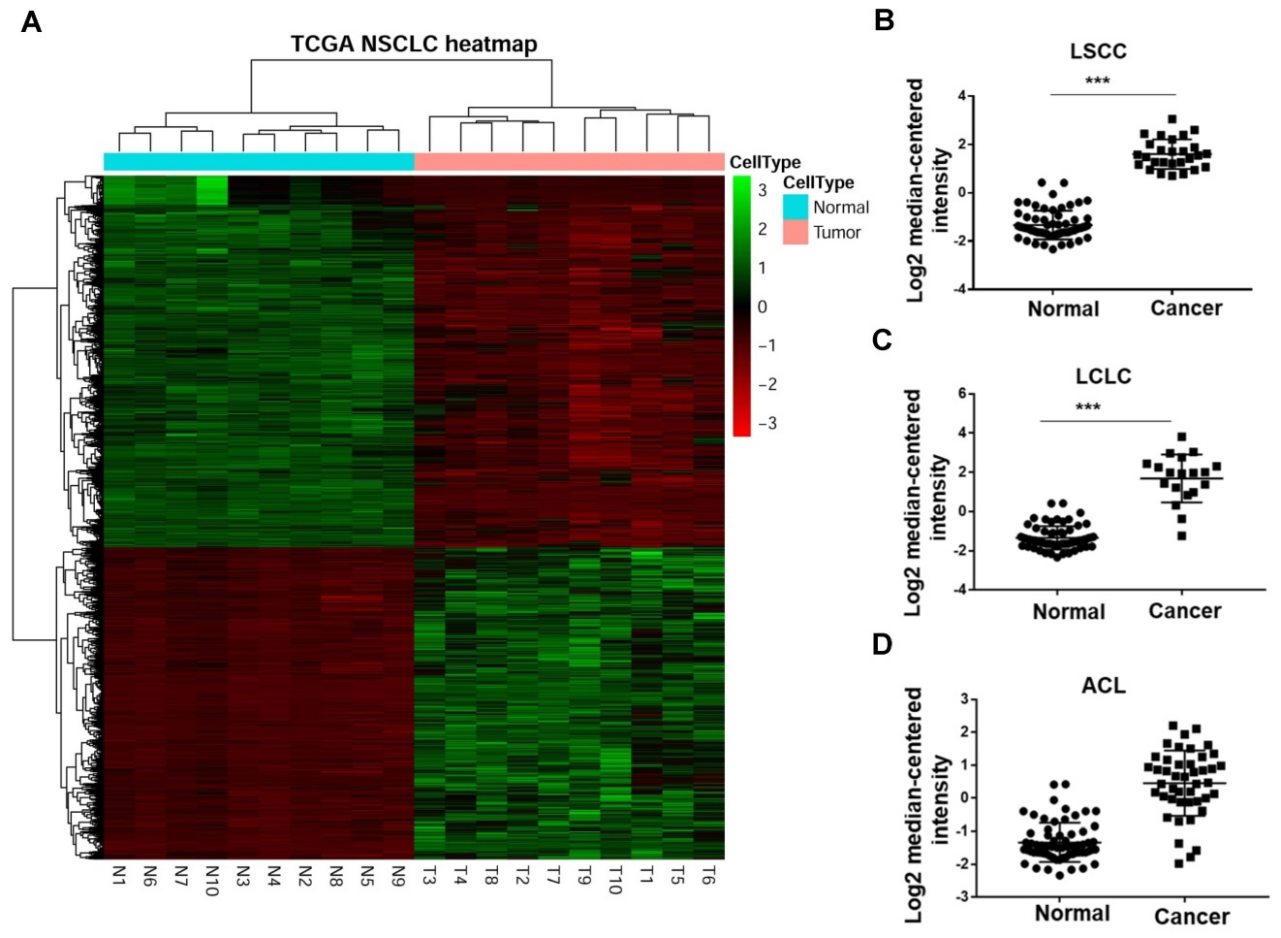
GINS2 was overexpressed in non-small-cell lung cancer (NSCLC) in the Cancer Genome Atlas database (TCGA). (A) Heat map from the TCGA database showing up- and downregulation of selected NSCLC-related genes. (B) (C) (D) GINS2 mRNA expression was measured in lung squamous cell carcinoma (LSCC), large cell lung carcinoma (LCLC) and adenocarcinoma of the lung (ACL) compared to paracancerous tissue (PCT). Data are expressed as the mean±SEM, n=89. ***P<0.001 vs. control.

In a previous study, Han and his colleagues^19^ performed RNA sequencing (RNA-Seq) to compare the transcriptomes of NSCLC and paracarcinoma tissue (PCT) to investigate gene expression. They enrolled 88 male patients (mean age, 61.2 years) with NSCLC, 54 patients with adenocarcinoma and 34 patients with squamous cell carcinoma. The heat map revealed that GINS2 was highly expressed in NSCLC patients (Figure 1A). Moreover, we compared GINS2 expression in lung squamous cell carcinoma (LSCC), large cell lung cancer (LCLC) and adenocarcinoma of the lung (ACL). The results showed that GINS2 was highly expressed in LSCC, LCLC and ACL patient lung tissues (Figure 1B, C and D).

**Figure 2:**
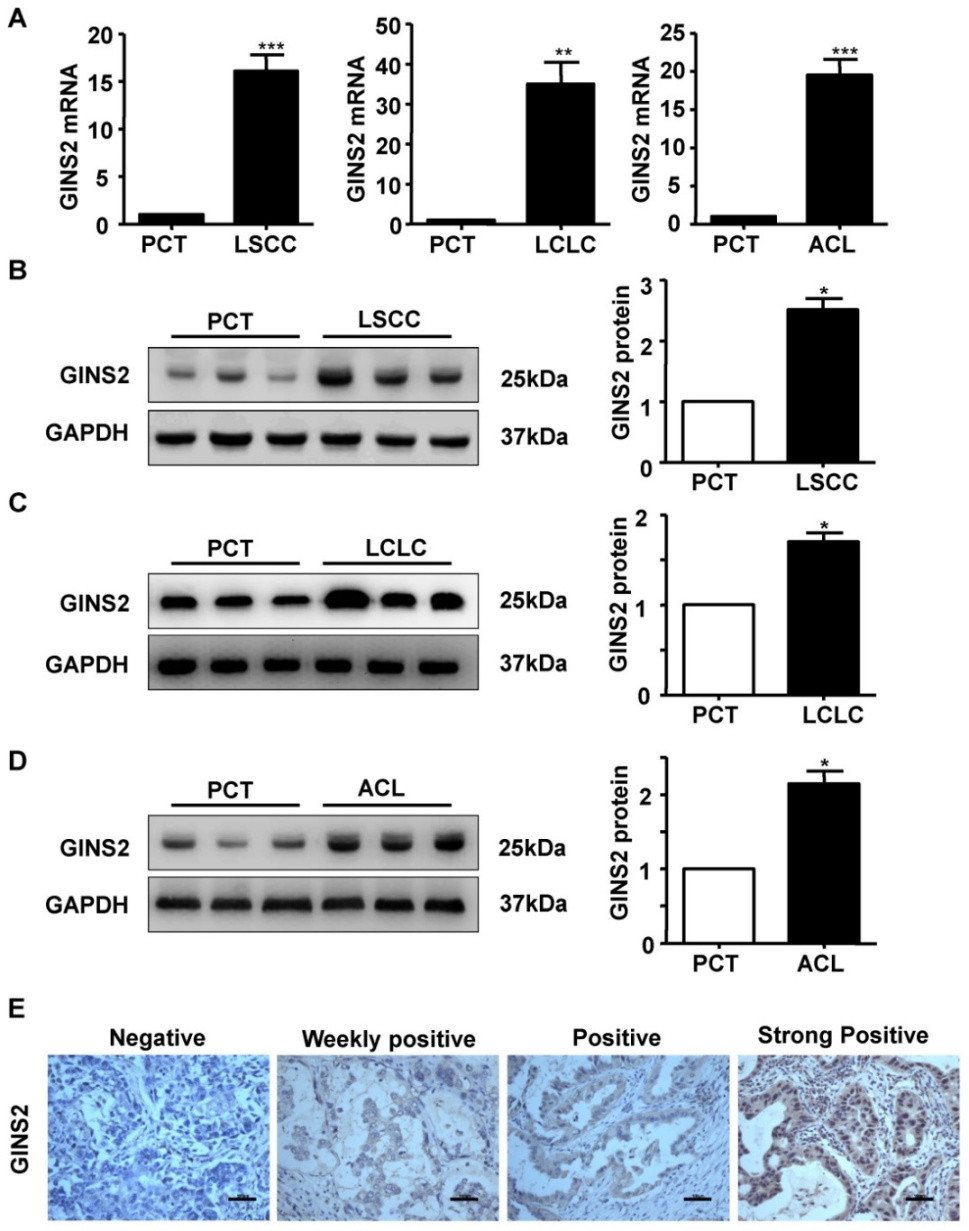
GINS2 was overexpressed in NSCLC tissues compared with PCTs. (A) GINS2 mRNA expression was determined by RT-PCR in LSCC, LCLC and ACL tissues and compared to that in PCTs. (B) (C) (D) Protein expression of GINS2 was determined by Western blotting in LSCC, LCLC and ACL compared to PCT. GAPDH was used as a loading control. Samples were pooled from 6 patients in each of the indicated groups. (E) GINS2 protein expression was measured by immunohistochemical staining. Data were expressed as the mean±SEM, n=6. *P<0.05, **P<0.01, ***P<0.001 vs. PCT.

In addition, we measured the mRNA and protein expression of GINS2 in LSCC, LCLC and ACL cancer tissues compared to PCT by RT-PCR and Western blotting. The results showed that GINS2 mRNA and protein expression were highly expressed in cancer tissue (Figure 2A, B, C and D), indicating that GINS2 was related to the occurrence and development of NSCLC.

The expression status of GINS2 was determined in patients with lung adenocarcinoma and the PCT by immunohistochemistry. GINS2 expression was not detected in PCT (Figure 2E). Of all the cancer specimens examined, 29 (18.9%) were negative for GINS2, 93 (60.8%) were weakly positive for GINS2 expression, 25 (16.3%) were positive for GINS2 expression and 6 (3.9%) were strongly positive for GINS2 expression (Figure 2E).

### Knockdown of GINS2 suppressed lung cancer cell proliferation, growth and colony formation in vitro

**Figure 3:**
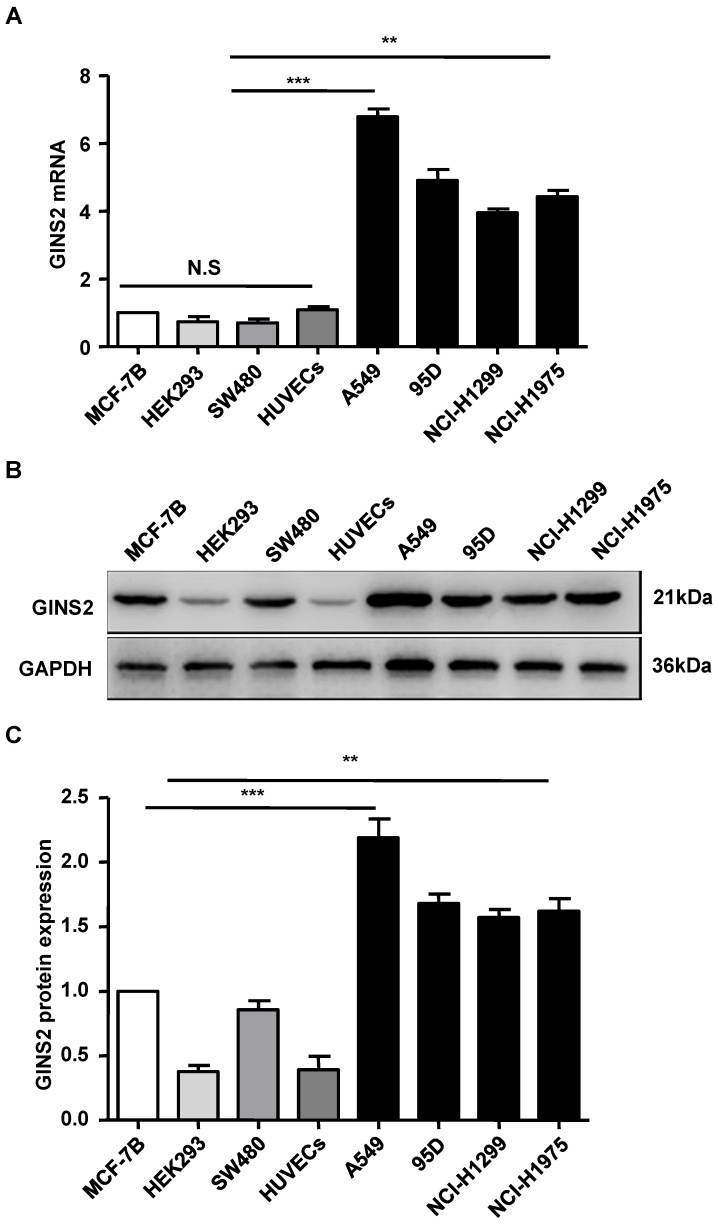
The expression pattern of GINS2 in cell lines. (A) GINS2 mRNA expression was measured by RT-PCR. (B) GINS2 protein expression was determined by Western blotting. (C) A representative blot and quantitation of data from six independent experiments were shown (GAPDH was used as loading control). The results were expressed as fold change compared with the NC cells. Data were expressed as the mean±SEM, *P<0.05, **P<0.01 vs. shCtrl.

We assessed the expression level of GINS2 via qRT-PCR. The results showed that GINS2 mRNA was highly expressed in A549 cells, moderately expressed in 95D, NCI-H1975 cells and NCI-H1299, and lowly expressed in HEK293 and HUVECs cells (Figure 3). A549 cells were selected and used for subsequent experiments.

**Figure 4:**
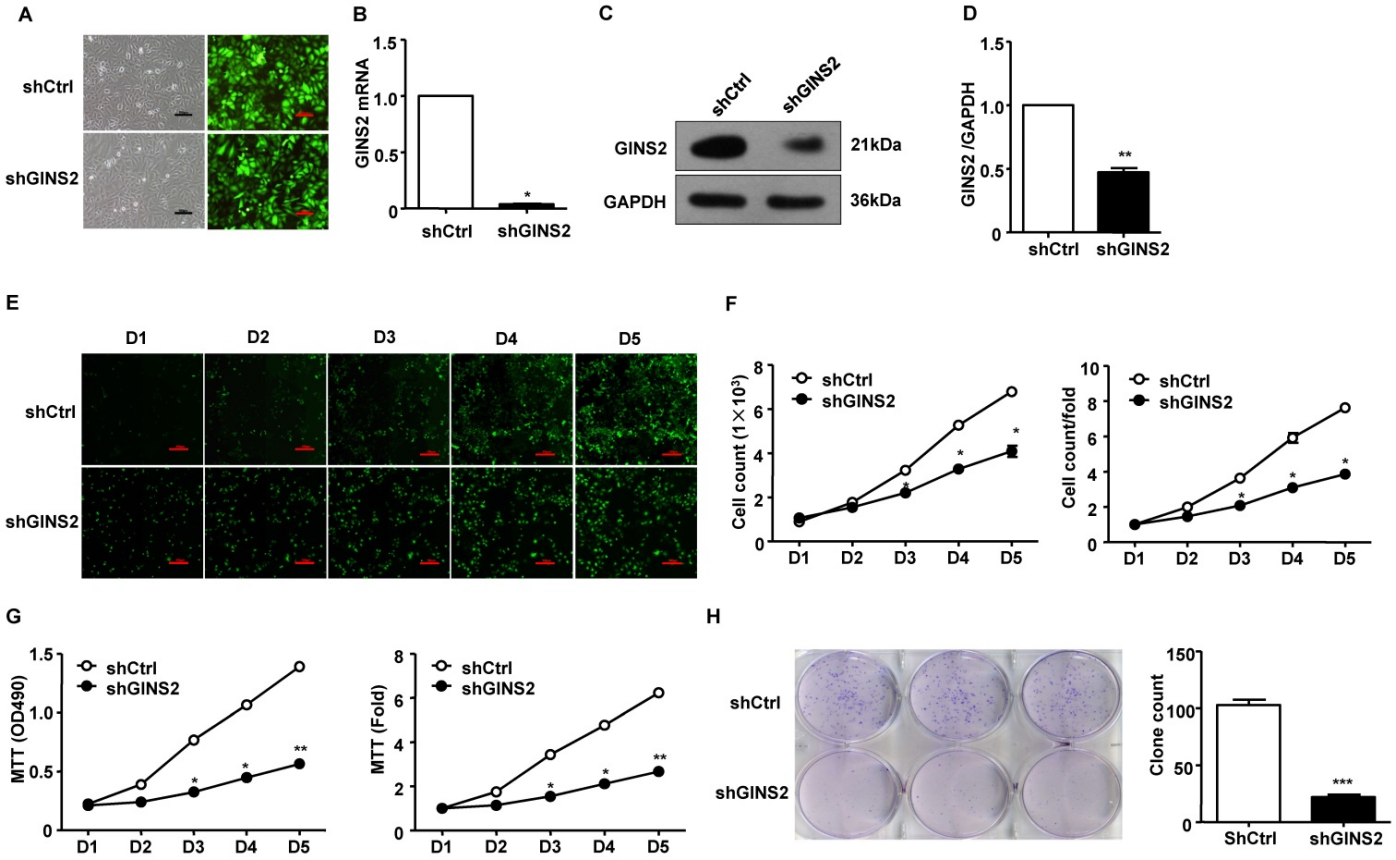
Knockdown of GINS2 inhibited A549 cell proliferation. (A) Fluorescence images of cells before and after transfection (×100). (B) GINS2 mRNA expression was significantly lower in the shGINS2 group than in the control group. (C) GINS2 protein expression was significantly lower in the shGINS2 group than in the control group. (D) A representative blot and quantitation of data from six independent experiments were shown (GAPDH was used as loading control). (E) A549 cell growth was measured every day for 5 days. Fluorescence images of cells were collected every day (×100). (F) A growth curve was generated to demonstrate the A549 cell growth rate in the shGINS2 and control groups. (G) The proliferation of A549 cells was significantly inhibited in the shGINS2 group compared to the control group. (H) GINS2 knockdown significantly reduced colony formation in A549 cells, as assessed by colony formation assay. Data were expressed as the mean±SEM, n=6. *P<0.05, **P<0.01 vs. shCtrl.

Second, we knocked down GINS2 gene expression in A549 cells. In order to evaluate the transfection efficiency of shCtrl and shGINS2, we observed the morphological characteristic of GFP protein in A549 cells after transfection of 72 h by inverted fluorescence microscope. The results showed that the transfection efficiency reached 70% after 72h (Figure 4A). In addition, the mRNA and protein expression levels of GINS2 in the shGINS2 group were significantly lower than those in the shCtrl group (Figure 4B, C and D).

We counted the cells every 5 days in the shCtrl and shGINS2 groups to detect cell growth. We found that knockdown of GINS2 significantly inhibited the cell growth ratio (Figure 4E and F). Second, we measured the cell proliferation of shGINS2 cells using the MTT assay. As shown in Figure 4G, after silencing GINS2 in A549 cells, the viable cell number increased significantly compared with that in the shCtrl group on days 4 and 5. Hence, knockdown of GINS2 inhibited cell proliferation (Figure 4G). Furthermore, we detected cell growth after transfection of shGINS2 and shCtrl lentivirus using a colony formation assay (Figure 4H).

### Knockdown of GINS2 induced cell cycle arrest, apoptosis and migration

**Figure 5:**
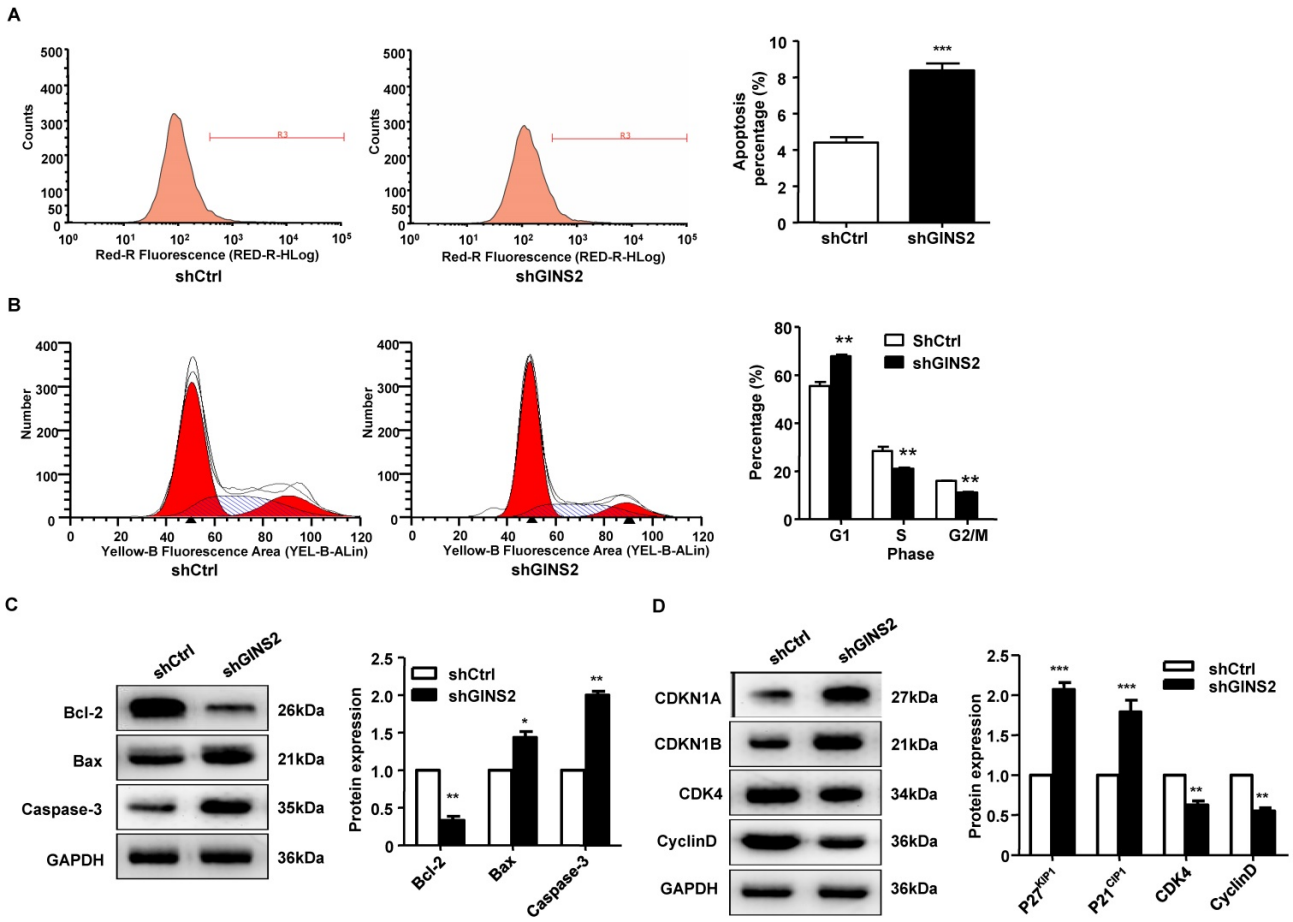
Knockdown of GINS2 promoted A549 cell apoptosis and proliferation stagnation. (A) A549 cells were transfected with shGINS2 and shCtrl lentivirus. (B) The proportion of cells in the G1 phase was significantly increased. (C) B-cell lymphoma-2 (Bcl-2) protein expression was increased significantly in the shGINS2 group compared to the control. Bcl-2 Associated X (Bax) and caspase-3 protein expression was decreased significantly in the shGINS2 group compared with the control. (D) Cyclin-dependent kinase inhibitor 1a (CDKI1A) and cyclin-dependent kinase inhibitor 1b (CDKI1B) protein expression was higher in shGINS2 cells than in control cells. Cyclin-dependent kinase 4 (CDK4) and cyclin D protein expression was decreased in shGINS2 cells compared with control cells. GAPDH was used as a loading control. Samples were pooled from 6 patients in each of the indicated groups. Data were expressed as the mean±SEM, n=6. *P<0.05, **P<0.01, *** *P*<0.001 vs. shCtrl.

To further understand the function of GINS2 in the cell cycle and apoptosis, fluorescence-activated cell sorting was used to detect the cell cycle and apoptosis rate. As shown in Figure 5A, the apoptosis rate was significantly higher in the shGINS2 group (8.38±0.29%) than in the control group (4.41±0.38%). Furthermore, after transfection with shGINS2 for 5 days, the percentages of A549 lung cancer cells in G2/M-phase and S-phase were significantly decreased, while the percentage of cells in G1-phase was dramatically increased (Figure 5B). These results indicated that cells with silenced GINS2 expression had a G2/M phase block.

Additionally, Bcl-2 protein expression was significantly decreased in the shGINS2 group compared with the control group. Bax and caspase-3 protein expression was significantly increased in the shGINS2 group compared with the control group (Figure 5C). On the other hand, CDKN1A and CDKN1B protein expression were significantly upregulated in the shGINS2 group compared to the control group (Figure 5D). CDK4 and cyclin D protein expression was decreased significantly in the shGINS2 group compared to the control group (Figure 5D).

**Figure 6:**
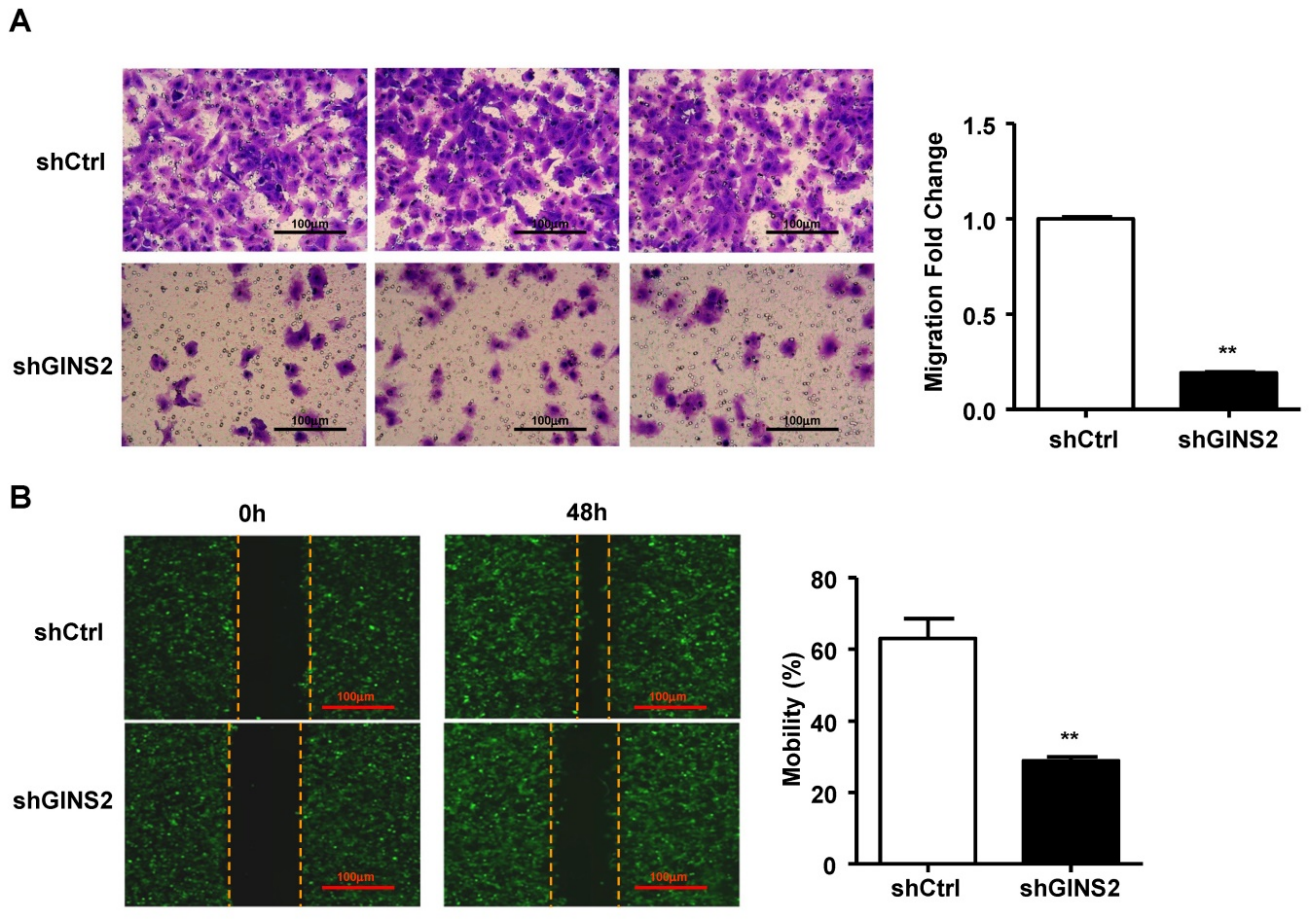
Knockdown of GINS2 inhibited the invasion of A549 cells. (A) A549 cells were analyzed by Transwell assay. (B) The cell scratch test was used to test cell mobility. Experiments were all performed in triplicate. Data were expressed as the mean±SEM, **P<0.01, vs. shCtrl.

In addition, we performed the Transwell assay and found that knockdown of GINS2 inhibited cell migration (Figure 6A). We also performed a cell wound scratch assay to assess cell mobility. We have outlined the cell boundary with dotted lines at the cell edge to clearly show the cell migration, the results showed that knockdown of GINS2 significantly reduced cell mobility (Figure 6B).

### Silencing of GINS2 inhibited tumorigenesis in nude mice

**Figure 7:**
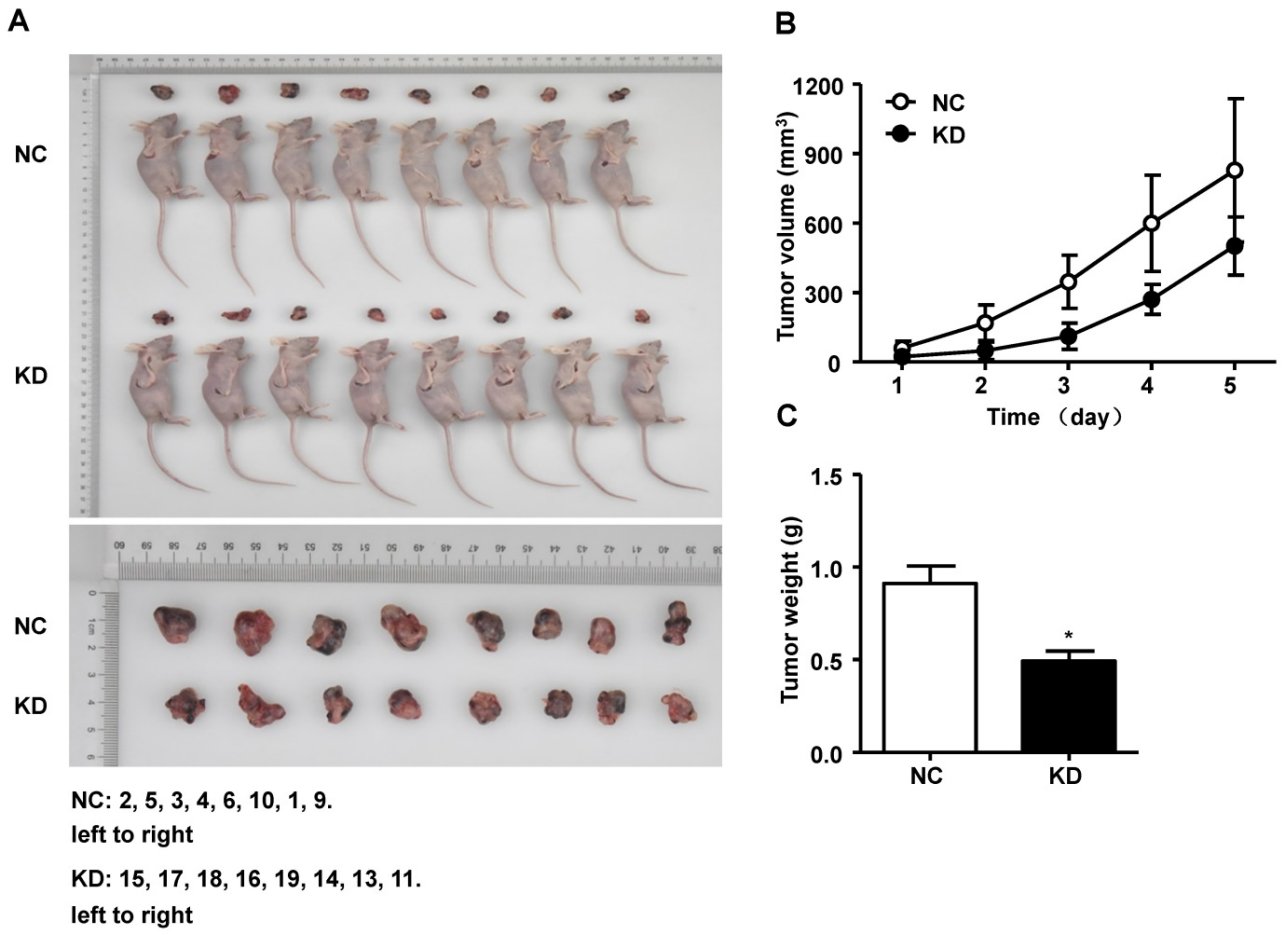
The effect of GINS2 knockdown on tumor progression in vivo. (A) The subcutaneous xenograft mouse models and representative images of tumors in mice. (B) Changes in tumor volume. (C) Changes in the tumor weight. NC, negative control. KD, knockdown. Data were expressed as the mean±SEM, **P<0.01, vs. shCtrl.

We performed an experiment to explore the effect of GINS2 on tumor progression in vivo. After euthanasia of mice, we measured the volume, weight and size of the tumors. As shown in Figure 7A, the tumor size was smaller in the shGINS2 group. Moreover, the tumor volumes in the shGINS2 group were significantly reduced compared to those in the control group (Figure 7B). Meanwhile, the weight of the tumors was significantly decreased in the shGINS2 group compared to the shCtrl group (Figure 7C).

### Elucidation of the mechanism of GINS2 in tumorigenesis in vitro

**Figure 8:**
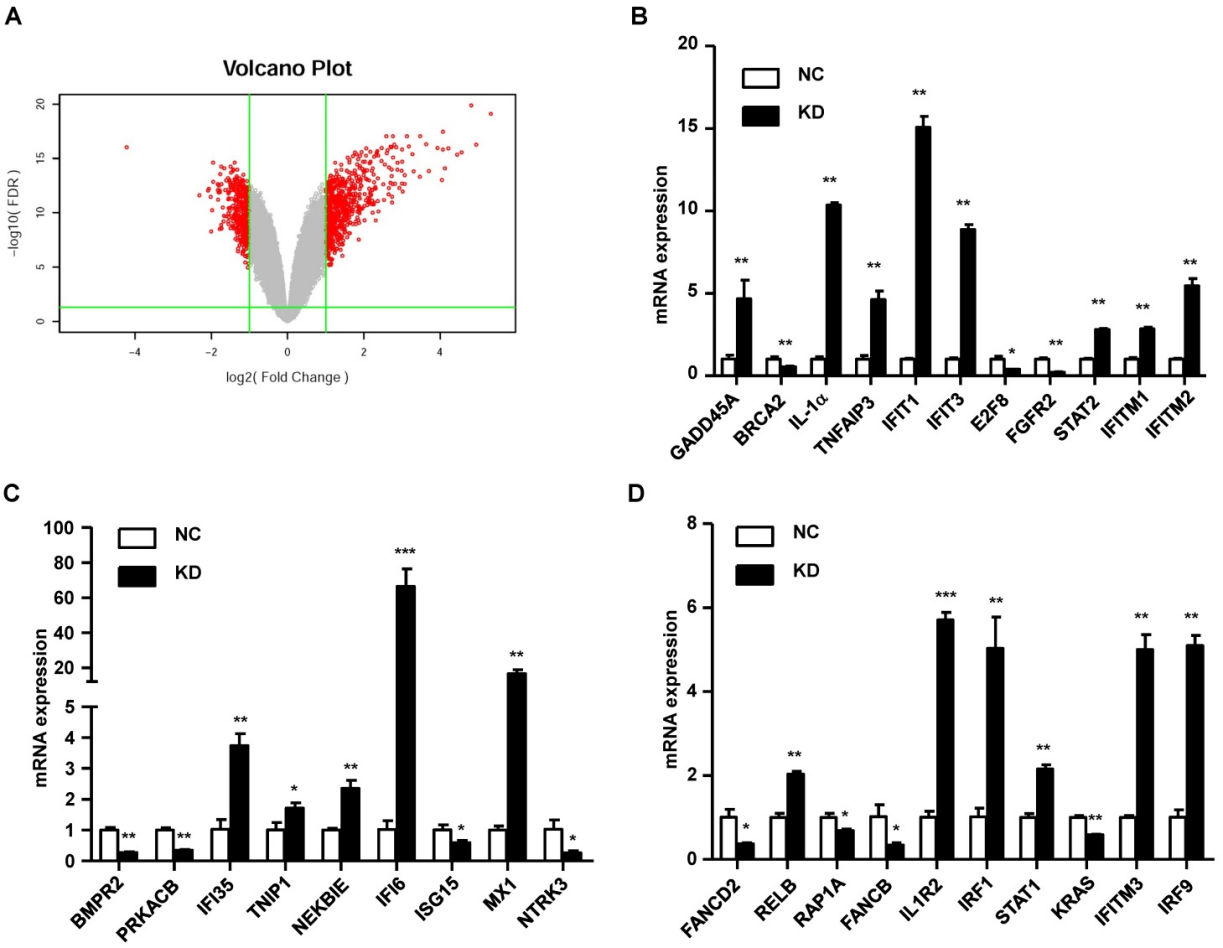
Mechanistic study of GINS2 knockdown in A549 cells. (A) Differentially expressed genes are shown as a volcano plot. (B) (C) (D) mRNA expression was assessed by RT-PCR. Data were expressed as the mean±SEM, **P<0.01, vs. shCtrl.

In our study, we found that knockdown of GINS2 in A549 cells inhibited cell proliferation and migration. To elucidate the mechanism of GINS2 in tumorigenesis in NSCLC, we performed RNA sequence analysis in A549 cells after knockdown of GINS2. We found 697 differentially expressed genes after knockdown GINS2 in A549 cells, and the results are shown as a volcano plot (Figure 8A). Then, we selected 30 genes and performed qRT-PCR to verify the RNAseq results and found that the results were consistent with the RNAseq analysis (Figure 8B, C and D).

**Figure 9:**
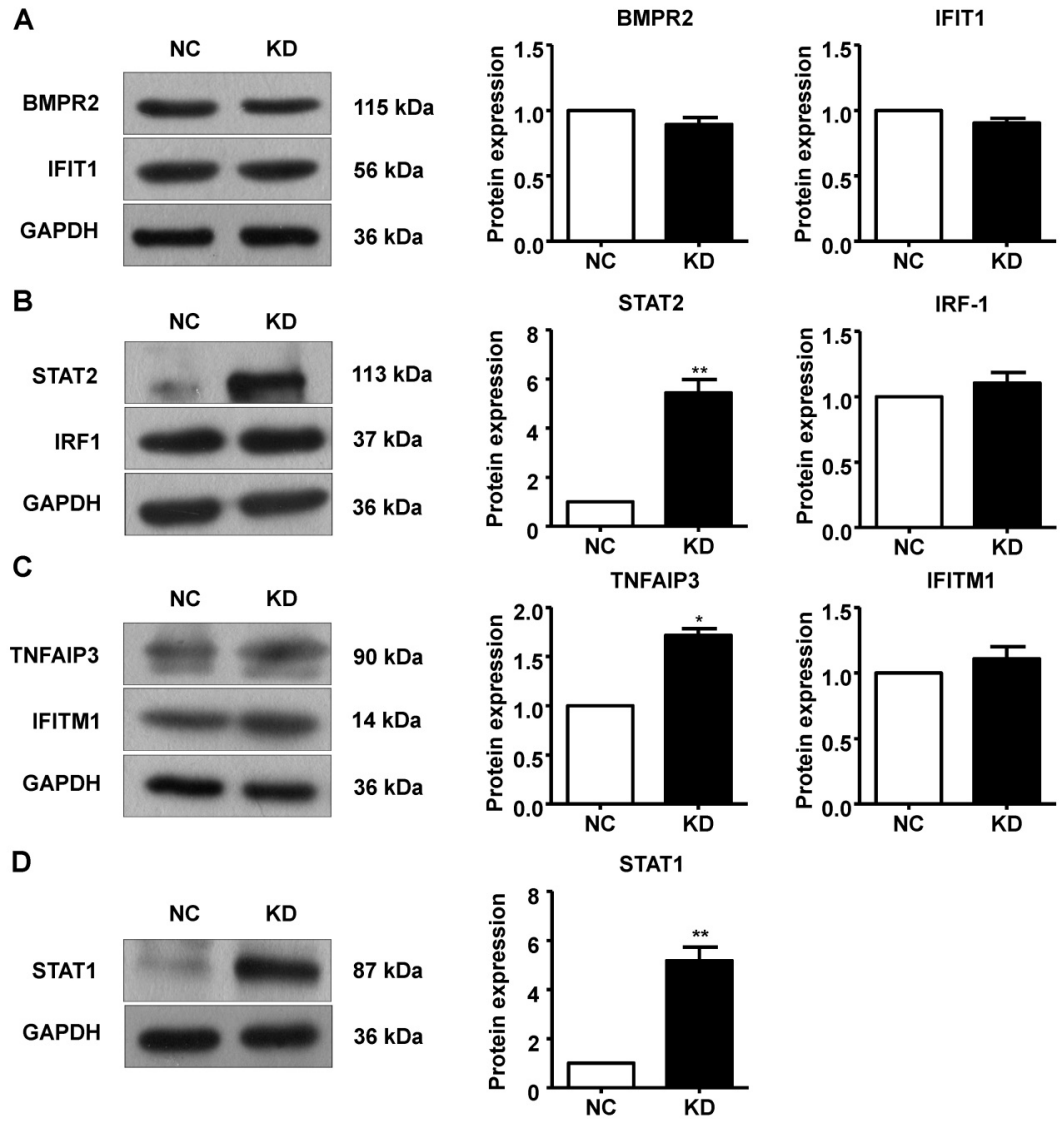
Knockdown of GINS2 unregulated the signal transducer and activator of transcription (STAT) signaling pathway. (A) Knockdown of GINS2 did not improve bone morphogenetic protein receptor type 2 (BMPR2) or interferon-induced protein with tetratricopeptide repeat 1 (IFIT1) protein expression. (B) Knockdown of GINS2 unregulated STAT1 protein expression. (C) Knockdown of GINS2 upregulated TNF alpha-induced protein 3 (TNFAIP3) protein expression. (D) Knockdown of GINS2 unregulated STAT1 protein expression. Experiments in this figure were all performed in triplicate. Data were expressed as the mean±SEM, **P<0.01, vs. shCtrl.

Additionally, we performed Western blotting to measure protein expression in A549 cells after GINS2 knockdown. BMPR2, IFIT1, IRF1 and IFITM1 protein expression was not changed in the GINS2 knockdown group compared to the control group (Figure 9A, B and C). STAT1, STAT2 and TNFAIP3 protein expression were all upregulated after GINS2 knockdown (Figure 9B, C and D).

## Discussion

This study combined TCGA database analysis and an animal model to investigate the role of GINS2 in NSCLC progression. In the TCGA database analysis, a significant correlation between increased GINS2 expression and carcinogenesis of lung cancer was observed. In addition, functional studies revealed that GINS2 promoted cell migration and proliferation and that STAT1 and STAT2 protein expression was elevated with GINS2 knockdown. Strikingly, the results showed the harmful effects of GINS2 in NSCLC progression in vivo. Moreover, RNA sequence analysis demonstrated that silencing GINS2 upregulated STAT1 and STAT2 protein expression in A549 cells. Hence, the expression level of GINS2 can influence physiological changes in NSCLC though the STAT signaling pathway.

Increased expression levels of GINS2 have been reported in many types of cancer, including thyroid cancer 20, epithelial ovarian cancer 21, cervical cancer 22, breast cancer 7, 23 and chronic myelogenous leukemia 11. In thyroid and epithelial ovarian cancer, GINS2 plays an essential role in cell apoptosis and proliferation and may be a potential biomarker for the diagnosis or prognosis of patients in the future 20, 21. Similarly, patients with higher GINS2 expression had shorter overall survival than patients with low GINS2 expression in early-stage cervical cancer 22. Previous studies considered the potential effects of GINS2 on cancer progression. Here, our results from TCGA data analysis confirmed the association between increased GINS2 expression and NSCLC progression. Additionally, the increased expression of GINS2 in tumor tissues observed in the NSCLC cohort supported the crucial role of GINS2 in NSCLC development.

We initially detected GINS2 mRNA expression in human lung cancer cell lines. Our results showed that A549 cells expressed high levels of GINS2, whereas 95D and NCI-H1975 cells expressed moderate levels. The reason for this difference may be the heterogeneity of cancer cells 24. In subsequent studies, we selected A549 cells to evaluate the effect of GINS2 and to determine its function in lung cancer.

The expression levels of apoptosis-related genes Bax and Bcl-2 were altered by GINS2 in a previous study, and these findings were consistent with ours 11. Tumorigenesis assays showed that the volume, size and weight of tumors in the shGINS2 group were significantly decreased compared to those in the control group, demonstrating that GINS2 knockdown inhibited tumor proliferation and growth in vivo.

To reveal the molecular mechanisms of GINS2 in NSCLC, we performed RNA sequencing to identify differentially expressed genes after GINS2 knockdown in A549 cells. Analysis of the cell signaling pathway showed that silencing GINS2 promoted STAT1 and STAT2 mRNA expression. STAT1 functions as a stimulator of growth factors, cytokines and hormones in several cell types 25. Previous research revealed that STAT1 acted as a tumor suppressor in esophageal squamous cell carcinoma and that constitutively activated STAT1 decreased cell growth and induced apoptosis 26. In another study, STAT2 knockout mice formed larger tumors than wild-type mice, indicating that STAT2 reduces tumor growth in a syngeneic tumor transplantation model 27. In addition to detecting gene expression, we found that GINS2 knockdown increased STAT1 and STAT2 protein expression and inhibited tumor migration and proliferation, which was consistent with a previous study. Furthermore, Huang et al reported that overexpression of TNFAIP3 inhibited migration and invasion in nasopharyngeal carcinoma 28. Our results showed that GINS2 knockdown increased TNFAIP3 protein expression, suggesting that TNFAIP3 may be involved in the effects of GINS2 on NSCLC proliferation and migration.

Taken together, based on previous studies, we determined the clinical significance of GINS2 in NSCLC and demonstrated that GINS2 knockout significantly impaired the proliferation and tumorigenicity of A549 cells. Furthermore, GINS2 inhibits the proliferation of NSCLC by inhibiting the STAT signaling pathway, which may be a potential biomarker for the diagnosis or prognosis of lung cancer.

## Abbreviations

Abs: antibodies
ACL: adenocarcinoma of the lung
ATCC: American Type Culture Collection
Bcl-2: b-cell lymphoma-2
BMPR2: bone morphogenetic protein receptor type 2
CDKN1A: cyclin dependent kinase inhibitor 1A
CDK4: cyclin dependent kinases 4
DMEM: Dulbecco's modified Eagle's medium
FDR: false discovery rate
FBS: fetal bovine serum
GAPDH: glyceraldehyde-3-phosphate dehydrogenase
GINS: GINS complex subunit
HEK293: human embryonic kidney 293
HUVECs: human umbilical vein endothelial cell
IFIT1: interferon induced protein with tetratricopeptide repeats 1
IFITM1: interferon induced transmembrane protein 1
IRF-1: interferon regulatory factor 1
KD: knock down
LCLC: large cell lung cancer
LSCC: lung squamous cell carcinoma
NC: negative control
NSCLC: non-small cell lung cancer
PBS: phosphate-buffered saline
PVDF: polyvinylidene difluoride
PCT: para-carcinoma tissue
RNA-Seq: RNA sequencing
STAT: signal transducer and activator of transcription
TCGA: The Cancer Genome Atlas
TNFAIP3: tumor necrosis factor alpha induced protein 3
