# Supplementary material for: GINS2 attenuates the development of lung cancer by inhibiting the STAT signaling pathway
Source: J Cancer. 2021 Jan 1;12(1):99–110. doi: 10.7150/jca.46744 (PMC7738824; doi:10.7150/jca.46744)
Supplement: Supplementary file 1 — Supplementary tables. [file jcav12p0099s1.pdf]

Supplementary table 1 The sequences of shRNA construction

**Gene**		**Sequences (5'-3')**
GINS2-shRNA	Sense	5'-CCGGGCGATTAACCTGAAACAAAGACTCGAGTCTTTGTTTCAGGTTAATCGCTTTTTG-3'
	Antisense	5'-AATTCAAAAAGCGATTAACCTGAAACAAAGACTCGAGTCTTTGTTTCAGGTTAATCGC-3'
Scramble-shRNA	Sense	5'-GATCCGTGGCATGCGTAGAAGGCAACTCGAGATTGCCTTCTACGCATGCCATTTTTC-3
	Antisense	5'-TCGAGAAAAAGTGGCATGCGTAGAAGGCAACTCGAGATTGCCTTCTACGCATGCCAG-3

Supplementary table 2 The sequences of qRT-PCR primers

Gene	Forward	Reverse	Products (bp)
GAPDH	TGACTTCAACAGCGACACCCA	CACCCTGTTGCTGTAGCCAAA	121
GINS2	CAGAAATGTCGCCTGCTCC	GGATTTCGTCTGCCTTCG	153
GADD45A	GAGAGCAGAAGACCGAAAGG	CAGCAGGCACAACACCAC	152
BRCA2	CACCCACCCTTAGTTCTACTGT	CCAATGTGGTCTTTGCAGCTAT	250
IL1A	AGATGCCTGAGATACCCAAAACC	CCAAGCACACCCAGTAGTCT	147
TNFAIP3	CCACGATGCTCAGGTTTG	TCCCTTTCTCAGCCAAGAC	222
IFIT1	GCGCTGGGTATGCGATCTC	CAGCCTGCCTTAGGGGAAG	96
IFIT3	AAAAGCCCAACAACCCAGAAT	CGTATTGGTTATCAGGACTCAGC	132
E2F8	AAGTACGCCGAGCAGATTATG	ATGTCTGGGTGTCCATTTGGG	128
FGFR2	GGTGGCTGAAAAACGGGAAG	AGATGGGACCACACTTTCCATA	104
IFITM1	TCCGTGAAGTCTAGGGACAG	GTCACAGAGCCGAATACCAG	152
STAT2	GGTTTGATTTGGGACTTTGGT	CTGCTGGTTCTGAAGGTTTGG	267
BMPR2	ACGGGTATCTTTTGTTGGTGT	TGTCAGCTTTCATAGTGGCATC	213
PRKACB	GATTGGTGGGCATTAGGAGTG	TCTGAACTGAAGTGGGATGGG	134
IFI35	TGAGCAGGTGCTGCAACAAAAG	CCACTCCATCCCTAGCAAACCC	299
IFITM2	ATGAACCACATTGTGCAAACC	CATAGCCACTTCCTGCTCCTC	90
TNIP1	CCTCTTCCATCTGCCGGAAT	TCTCATCCAGCTGAGGCTCT	258
NFKBIE	TTACCCATGTTGGGTCAGCC	AACGGTGTTTCAGGGTCCTC	140
IFI6	CTCGCTGATGAGCTGGTCT	GATACTTGTGGGTGGCGTAG	149
ISG15	CAGCTCCATGTCGGTGTCA	AGGTTCGTCGCATTTGTCC	184
MX1	GGTCAGTTACCAGGACTACGAG	GGTTATGCCAGGAAGGTCTATT	181

NTRK3	AAGCCCACCCACTACAACAAT	TGGGACTCACTTCGTCAAACA	148
FANCD2	CACTGACCAGGCATACTTT	TCTAGGAGCGGCATACATT	268
RELB	CAGCCTCGTGGGGAAAGAC	CCAGCGTTGTAGGGGTCAA	171
RAP1A	TTGAGGCGGTCTAACTACCC	CAACGAACATACATCATCCC	300
FANCB	CAACTTATGGATTCAGGTGGAG	TTTCCCATTTAGCAGCAACC	109
IL1R2	CCTGGAAGATGCTGGCTATTAC	CGGTTCCCAGAAACACCTTAC	200
IRF1	GATGCTTCCACCTCTCACC	TGCTCCACCTCCAAGTCC	201
STAT1	GGCACCAGAACGAATGAGG	CCACAACGGGCAGAGAGG	124
KRAS	GGAGGGCTTTCTTTGTGTATTTG	TGTCTTGTCTTTGCTGATGTTTC	225
IFITM3	TCGTCTGGTCCCTGTTCAA	CCAACCATCTTCCTGTCCC	100
IRF9	CAGTAGTTGTCCGTGATAATCG	GTGCTTGGCTTTAGAGTTGG	205
